# TRIM59 Protects Mice From Sepsis by Regulating Inflammation and Phagocytosis in Macrophages

**DOI:** 10.3389/fimmu.2020.00263

**Published:** 2020-02-18

**Authors:** Zheng Jin, Zhenhua Zhu, Shanshan Liu, Yuyang Hou, Mengyan Tang, Pei Zhu, Yuan Tian, Dong Li, Dongmei Yan, Xun Zhu

**Affiliations:** ^1^Department of Immunology, College of Basic Medical Sciences, Jilin University, Changchun, China; ^2^Department of Orthopaedic Trauma, The First Hospital of Jilin University, Changchun, China; ^3^Key Laboratory for Molecular Enzymology and Engineering, Ministry of Education, Jilin University, Changchun, China

**Keywords:** TRIM59, macrophages, sepsis, inflammation, phagocytosis

## Abstract

Sepsis is associated with bacterial invasion and inflammation and has a high mortality rate. Previous studies have demonstrated that tripartite motif 59 (TRIM59) was involved in NF-κB signaling and could promote phagocytosis of macrophages, but the role of TRIM59 in sepsis is still unknown. In our study, we found that TRIM59 was downregulated in lipopolysaccharide (LPS)-stimulated bone marrow-derived macrophages (BMDMs). In the cecal ligation and puncture (CLP) sepsis mice model, the mortality of *Trim59*^*flox*/*flox*^*Lyz-Cre* (*Trim59*-cKO) mice was higher, the immune cell infiltration and damage of liver and lung were more severe, and bacteria burden was increased. We also found that TRIM59 altered the production of pro-inflammation cytokines, as well as macrophage phagocytosis ability. Further analysis indicated that NF-κB signal pathway and Fcγ receptors might be involved in these regulations. Our study demonstrated for the first time that TRIM59 protects mice from sepsis by regulating inflammation and phagocytosis in macrophages.

## Introduction

Sepsis is a life-threatening syndrome that affects the health of millions of patients, especially hospitalized patients. Sepsis occurs when there is a dysregulated host response to infection and can cause severe organ dysfunction (1). According to previous research, the mortality rate of sepsis is as high as 25% (2). Sepsis certainly imposes significant global health costs due to its high incidence and mortality rates. Therefore, it is of utmost importance to determine the pathogenesis of sepsis and its mitigating factors.

Macrophages are an integral part of the innate immune response and play a key role in many diseases. Macrophages have multiple functions, including: (1) production of inflammatory cytokines, like interleukin-1 (IL-1), IL-6, and tumor necrosis factor-α (TNF-α); (2) phagocytosis of pathogens; and (3) presentation of antigens with major histocompatibility complex (MHC) molecules, cluster of differentiation (CD) 80 and CD86 (3). TNF, IL-1β, and IL-6 are important cytokines that mediate initial response of innate immune system to infections. TNF and IL-1β both activate endothelial cells and attract circulating polymorphonuclear white blood cells (PMNs) to the infection site. However, these cytokines also enter the bloodstream and cause fever and other systemic symptoms. IL-6 also stimulates liver production of acute phase reactants to stimulate the inflammatory response, and stimulates the transformation of bone marrow cells to produce more PMNs, both of which in turn increase inflammation (4). The pro-inflammatory cytokines produced by macrophages are harmful during sepsis. However, macrophages can consume large amounts of bacteria, which limits the damage that results from sepsis (5). Previous studies have demonstrated reduced expression of MHCII proteins on the surface of macrophages in sepsis patients, which resulted in T cells not activating effectively and aggravating the sepsis (6). Therefore, macrophages play an important role in the development of sepsis.

TRIM59 is a member of TRIM family, which is composed of a RING finger domain, a B-Box motif, and two coiled-coil regions from the N to the C-terminal. The TRIM protein family is also referred to as the RBCC protein family due to its highly conservative structure (7). TRIM proteins are involved in the innate immune response and recent studies have confirmed that TRIM family proteins could restrict retroviral infections and influence various signaling pathways such as IFN signaling pathway and TLR signaling pathway. Additionally, TRIM family proteins participate in the regulation of cytokine gene transcription (8, 9). TRIM59 is upregulated in various cancers and promotes the development of tumors (10). TRIM59 is downregulated in LPS-stimulated macrophage cell lines and can regulate the innate immune response through the NF-κB and IRF-3/7-mediated signal pathways by interacting with evolutionarily conserved signaling intermediates in the Toll pathway (ECSIT) (11, 12). However, the role of TRIM59 in sepsis has not been explored.

In our study, we demonstrated that the expression of TRIM59 was significantly downregulated in LPS-stimulated bone marrow-derived macrophages (BMDMs). In *Trim59*-cKO mice, after CLP surgery, mortality, liver and lung damage, as well as bacterial burden were increased. Additionally, the expression of Fcγ receptors and phagocytosis were weakened, and the secretions of pro-inflammatory cytokines were upregulated related to NF-κB signaling pathway in *Trim59*-knocked out BMDMs. Therefore, we concluded that TRIM59 protected mice from sepsis by regulating inflammation and phagocytosis in macrophages.

## Materials and Methods

### Animals

Mice were bred under specific-pathogen-free (SPF) conditions and housed in temperature-controlled, air-conditioned facilities with 12 h/12 h light/dark cycles and unlimited access to food and water. *Trim59*^*flox*/*flox*^ (Cyagen Biosciences Inc., China) mice with floxed alleles were bred with *Lyz-Cre* (Jackson Laboratories, USA) mice to generate *Trim59*-cKO mice. Both strains of transgenic mice came from a C57BL/6N genetic background. Control mice for all experiments were littermate *Trim59*^*flox*/*flox*^ mice lacking the Cre transgene. C57BL/6N mice were the source of wild-type macrophages. The structure and genotype identification of the mice were presented in Figure S1.

### Cell Culture

Bone marrow cells were removed from the femurs of 6–8-week old mice and cultured for 7 days in dulbecco's modified eagle medium (DMEM) (BI, Israel) supplemented with 10% fatal bovine serum (FBS) (BI, Israel) and 30% conditioned medium from the L929 fibroblast cell line. After 7 days, the cells were assessed using F4/80 (BD, USA) staining. BMDMs were cultured overnight prior to use.

### Sepsis Model

A cecal ligation and puncture (CLP) mice model as described previously (13) was used for this study. In brief, mice were anesthetized using an intraperitoneal injection of sodium pentobarbital (Sigma-Aldrich, USA). A midline incision was made, followed by externalization, and then the cecum was ligated (1 cm from the apex) and punctured with a 22G needle. Next, a small amount of fecal mass from the punctured cecum was gently squeezed out to ensure patency of punctures, the cecum was relocated, and 4/0 sutures were used to close the peritoneum and skin. Sham-operated mice underwent only incision and cecum exteriorization.

### Measurement of Cytokines in Peripheral Blood and Supernatants

Twenty-four hours following CLP surgery or sham surgery, mice were again anesthetized using sodium pentobarbital (200 mg/kg). The serum was separated using centrifugation at 3,000 g for 15 min at 4°C and then stored at −80°C. The supernatants of BMDMs were collected after stimulation with 0.2 μg/ml LPS for 0, 6, 12, 18, 24, 48 h, or with 0, 0.1, 0.2, 0.5, 1, 5 μg/ml LPS for 24 h. The concentrations of IL-1β, TNF-α, IL-6, IL-10, and TGF-β1 in the blood serum and supernatants were determined using Elisa kits (eBioscience, USA) according to the manufacturer's instructions. All samples were measured in triplicate.

### Measurement of NO Levels in Peripheral Blood and Supernatants

Serum and supernatants were collected as described. The levels of NO were measured using a separate kit (Beyotime Biotechnology, China) according to the manufacturer's instructions.

### Measurement of ALT and AST Levels in Peripheral Blood

Serum was collected as described. The levels of AST and ALT in serum were determined using common biochemical kits (JianCheng, China).

### Histological Analysis

Twenty-four hours following CLP surgery or sham surgery, animal tissue samples were fixed in a 10% neutral buffered formalin solution for 24 h, embedded in paraffin, and sectioned to a thickness of 5 μm. Samples were subsequently stained with hematoxylin and eosin. All samples were photographed and examined immediately using OLYPUS (BX53, Japan). Liver damage scoring criteria: 1 point, normal tissue; 2 points, focal fragmentation necrosis of hepatocytes, 2–3 fold infiltration of inflammatory cells, and hemorrhage <30%; 3 points, hepatocyte continuous necrosis <50%, 3–10 fold infiltration of inflammatory cells, and hemorrhage at 30–50%; 4 points, hepatocyte continuous necrosis >50%, inflammatory cells infiltration >10-fold, and hemorrhage >50% (14). Lung damage scoring criteria: 0 point, normal tissue; 1 point, focal interstitial hyperemia and inflammatory cells infiltration; 2 points, diffuse interstitial hyperemia and inflammatory cells infiltration; 3 points, alveolar wall capillary dilatation and hyperemia, alveolar wall widening (inflammatory cell infiltration/fibrosis); 4 points, alveolar wall capillary dilation and hyperemia, alveolar wall widening (inflammatory cell infiltration/fibrosis), alveolar cavity exudation, alveolar consolidation, accompanied by infiltration of inflammatory exudates in bronchial cavity (14). Lung inflammation scoring criteria: 0 point, normal tissue; 1 point, a few cells; 2 points, a ring of inflammatory cells 1 cell layer deep; 3 points, a ring of inflammatory cells 2–4 cells deep, and 4 points, a ring of inflammatory cells >4 cells deep (15).

### Flow Cytometry Analysis

Peripheral blood, spleens, and mesenteric lymph nodes (mLN) were collected 24 h after CLP surgery or sham surgery to obtain single cells for flow cytometry. Cells were stained for 30 min at 4°C with the following antibodies: CD45-Percp, CD3-FITC, CD4-Percp, CD8-APC, B220-PE, CD11c-APC, CD11b-FITC, F4/80-PE, and Ly6G-PE. Following incubation, red blood cells were lysed and washed 3 times with a FACS buffer before collecting data. Immunophenotyping analysis of the BMDMs was conducted using a flow cytometry technique. Briefly, 100 μl of cell suspension was incubated for 5 min with 5 μl of Fc blocker (Innovex, USA). Then, 1 μl of monoclonal antibodies (CD11b-FITC, F4/80-PE, F4/80-FITC, MHCII-FITC, CD80-PE, CD80-APC, CD16/32-Percp-cy5.5, and CD64-APC, BD, USA) were added and incubated at 4°C for 30 min and washed 3 times with the FACS buffer. All samples were analyzed using a FACSCalibur flow cytometer (BD, USA).

### Bacteria Count

Mouse peripheral blood and peritoneal fluid were collected 24 h following CLP. Blood agar plates were coated with a 5 μl dilution. Bacteria were counted following incubation at 37°C for 24 h and the count was calculated as CFU per whole blood.

### Quantitative Real-Time Polymerase Chain Reaction

BMDMs were stimulated using LPS. Total RNA was extracted using a Trizol reagent (Takara, Japan), and reverse-transcribed into cDNA using a cDNA Synthesis Kit (Takara, Japan). Quantitative real-time polymerase chain reaction (qRT-PCR) was performed using an Agilent Mx3000P machine and the TransStart Green qPCR SuperMix (Takara, Japan), according to the manufacturer's instructions. Each sample was conducted in triplicate and the gene expression levels were calculated relative to the amount of GAPDH using the 2^−ΔΔCT^ method. The primer sequences for the tested genes were listed in Table 1.

**Table 1:** List of primer sequences.

**TRIM59**	**Forward**	**5′-GCTTCTACTGGCATAGAATCCTTAC-3′**
	Reverse	5′-ACATCTGGGTGGTCTTCTTGCT-3′
GAPDH	forward	5′-GACTTCAACAGCAACTCCACTC-3′
	Reverse	5′-TAGCCGTATTCATTGTCATACCAG-3′
iNOS	forward	5′-CAAGCACCTTGGAAGAGGAG-3′
	reverse	5′-AAGGCCAAACACAGCATACC-3′
TNF-α	forward	5′-GTCAACCTCCTCTCTGCCAT-3′
	reverse	5′-CCAAAGTAGACCTGCCCAGA-3′
IL-1β	forward	5′-GCAACTGTTCCTGAACTCAACT-3′
	reverse	5′- ATCTTTTGGGGTCCGTCAACT-3′
IL-6	forward	5′-CCAAGAGGTGAGTGCTTCCC-3′
	reverse	5′- CTGTTGTTCAGACTCTCTCCCT-3′
IL-10	forward	5′- CTTACTGACTGGCATGAGGATCA-3′
	reverse	5′- GCAGCTCTAGGAGCATGTGG-3′
TGF-β	forward	5′- CTCCCGTGGCTTCTAGTGC-3′
	reverse	5′- GCCTTAGTTTGGACAGGATCTG-3′

### Western Blot Analysis

After being treated with LPS, the total protein level of the BMDMs was collected using RIPA (Beyotime, China) and protease inhibitor (Roche, USA). Additionally, the cytoplasmic and nuclear protein fractions were extracted by using the ProteinExt Mammalian Nuclear and Cytoplasmic Protein Extraction Kit (TransGen Biotech, China). The levels of TRIM59 (Abcam, USA), p65 (CST, USA), p-p65 (CST, USA), IκB (CST, USA), p- IκB (CST, USA), IKKα (CST, USA), IKKβ (CST, USA), p-IKKα/β (CST, USA), ECSIT (ABclonal Biotechnology, China), MAP3K1 (ABclonal Biotechnology, China), AKT (CST, USA), p-AKT^Ser473^ (CST, USA), p-AKT^Thr308^ (CST, USA), PI3K (CST, USA), p-PI3K (CST, USA), GAPDH (Proteintech, China), and Histone-3 (H3, Proteintech, China) were measured using Western blot analysis.

### Transfection of *E. coli* BL21 With Plasmid

We placed 100 μl *E. coli* BL21 (TIANGEN, China) into an ice bath and added 1 ng Pet-14b-EGFR plasmid (Public Protein/Plasmid Library, China), which was followed by a water bath at 42°C for 60 s, followed by an ice bath for 3 min, and finally added 900 μl of LB medium. After mixing, we placed the mixture in a shaking bed at 37°C and incubated for 45 min at 150 rpm/min. After shaking, 100 μl of liquid containing kanamycin was evenly coated on the LB solid medium, and the plate was inverted and cultured overnight at 37°C. On the following day, the monoclonal bacteria were selected and cultured in the LB medium containing kanamycin at 200 rpm/min in a shaking bed at 37°C for 12 h.

### Phagocytosis Assays

The BMDMs were stimulated with 0.2 μg/ml LPS for 24 h. *E. coli* were coated with mouse IgG (Bioss, China, 1 mg/ml) for 1 h at 37°C, rinsed, reconstituted in DMEM, and then added to the BMDMs at a 20:1 ratio to synchronize binding and internalization. After 30 min at 37°C, non-adherent *E. coli* were removed using cold PBS, and cells were fixed in 3.7% formalin. Fifteen fields were photographed using bright field and epifluorescence microscopy. Phagocytosis rate and the phagocytosis index were calculated according to the following formulas: phagocytosis rate = the number of macrophage phagocytosis of *E. coli* in 100 cells /100 × 100%; Phagocytosis index = the total number of phagocytic *E. coli* in 100 cells /100 × 100%.

### Statistical Analysis

Data are presented as Means ± SEM from at least triplicate samples. Besides, each experiment was repeated at least three times. Comparisons were statistically tested using Student's *t* test. *P* < 0.05 was considered to be statistically significant.

## Results

### TRIM59 Was Suppressed When Stimulated With LPS in BMDMs

**Figure 1:**
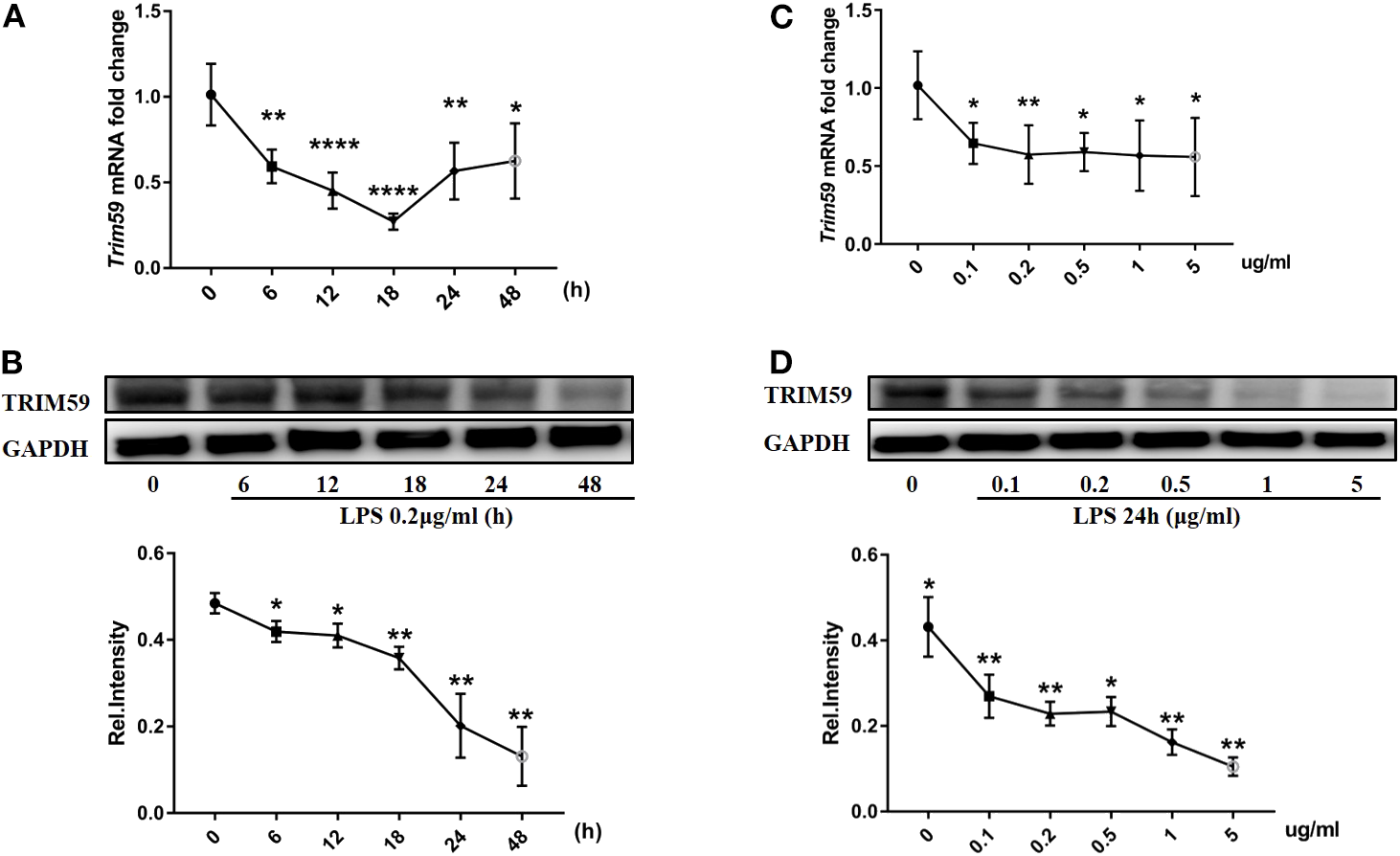
TRIM59 was suppressed when stimulated with LPS in BMDMs. **(A,B)** BMDMs from C57BL/6N mice were stimulated with LPS (0.2 μg/ml) for the indicated durations. *Trim59* mRNA levels were determined using qRT-PCR **(A)**, TRIM59 protein levels were determined using western blot analysis **(B)**; **(C,D)** BMDMs from C57BL/6N mice were stimulated using LPS (24 h) for the indicated dose. *Trim59* mRNA levels were determined using qRT-PCR **(C)**, and TRIM59 protein levels were determined using western blot analysis **(D)**. Relative intensities were quantitated by densitometry using ImageJ and normalized by GAPDH levels. Data are presented as Means ± SEM, *p < 0.05, **p < 0.01, ****p < 0.0001.

The purity of BMDMs from C57BL/6N (wild type) mice was identified to be 93.5%. We also divided and cultured BMDMs from *Trim59*^*flox*/*flox*^ and *Trim59*-cKO mice, and the purities of BMDMs was tested to be 94.7 and 96.6%, respectively (Figure S2). Besides, there was no difference in the number of BMDMs from *Trim59*^*flox*/*flox*^ and *Trim59*-cKO mice (data not shown). To confirm the expression of TRIM59 in the inflammatory environment of macrophages, we simulated BMDMs from C57BL/6N mice with LPS, and the results showed that TRIM59 expression levels were decreased regardless of the stimulating time and dose (Figure 1).

### Myeloid-Derived TRIM59 Positively Regulated Host Response to Sepsis

**Figure 2:**
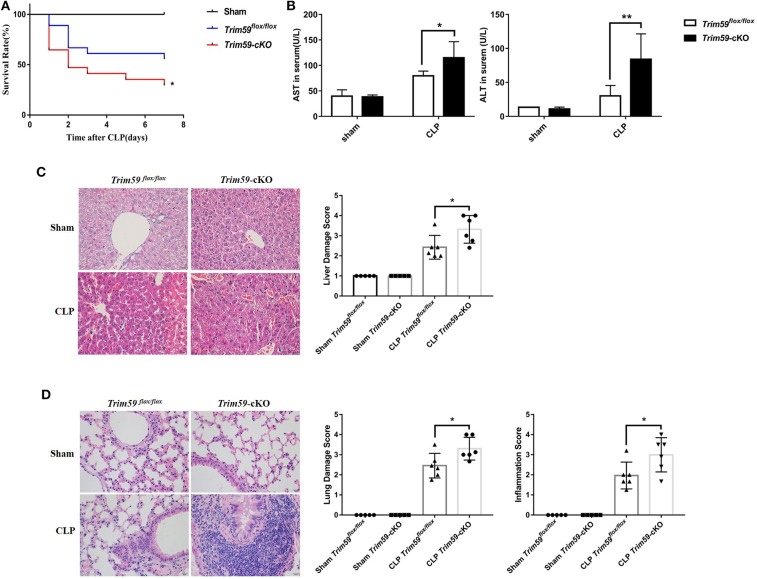
Deletion of myeloid-derived TRIM59 in macrophages reduced host response to sepsis. **(A)** Sepsis models were established in *Trim59*^*flox*/*flox*^ and *Trim59*-cKO mice using CLP. Survival was monitored until 7 days after the surgery. **(B)** AST and ALT were measured in the blood serum of *Trim59*^*flox*/*flox*^ and *Trim59*-cKO mice 24 h after surgery. **(C)** The results of the H&E staining of liver tissues from *Trim59*^*flox*/*flox*^ and *Trim59*-cKO mice after CLP for 24 h. **(D)** The results of the H&E staining of lung tissues from *Trim59*^*flox*/*flox*^ and *Trim59*-cKO mice after CLP for 24 h. Original magnification ×400, scale bar = 20 μm. Sham group, *n* = 5; CLP group, *n* = 6. Data are presented as Means ± SEM, *p < 0.05, **p < 0.01.

Following CLP surgery, control mice exhibited a 50% 7-days survival rate. In contrast, the survival rate among *Trim59*-cKO mice was only 30% (Figure 2A). The levels of ALT and AST were higher in the *Trim59*-cKO mice (Figure 2B). Further analysis showed that after CLP surgery at 24 h, *Trim59*-cKO mice revealed more obvious necrosis and bleeding areas in the liver (Figure 2C), and marked damage and inflammatory cell infiltration in the lung (Figure 2D). No significant damage was found in the heart, spleen, kidney, or brain (Figure S3). All results indicated that the loss of TRIM59 aggravated the host's response to sepsis.

### Deletion of Myeloid-Derived TRIM59 in Macrophages Increased the Infiltration of Neutrophils

**Figure 3:**
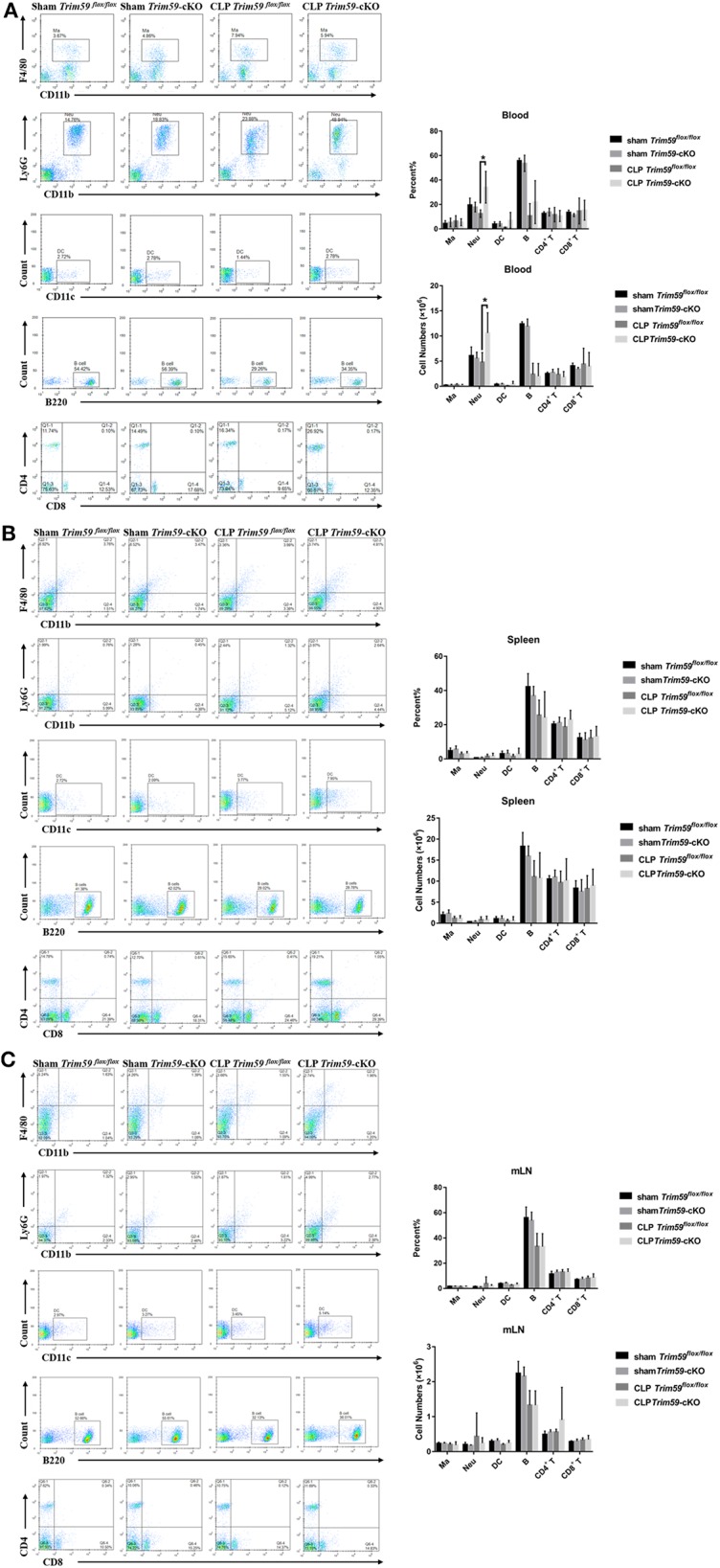
Deletion of myeloid-derived TRIM59 in macrophages increased the infiltration of neutrophils. Peripheral blood, spleen, and mLN were obtained from the sham or CLP group mice 24 h after surgery. Flow cytometric profiles and quantification of CD11b^+^ F4/80^+^ Ma, CD11 b^+^ Ly6G^+^ Neu, CD11c^+^ DC, B220^+^ B cells, CD4^+^ T cells and CD8^+^ T cells in peripheral blood **(A)**, spleen **(B)**, and mLN **(C)**. Sham group, *n* = 5; CLP group, *n* = 6. Data are presented as Means ± SEM, *p < 0.05.

To confirm the effect of TRIM59 deficiency on immune cells in mice with sepsis, we collected the peripheral blood, spleen, and mesenteric lymph node (mLN) from sham or CLP group mice 24 h after surgery. We found a large number of neutrophils infiltrated the peripheral blood of *Trim59*-cKO mice with sepsis, which indicates a more severe inflammatory response (Figure 3A). We also analyzed the percentages and absolute numbers of B cells, CD4^+^ T cells, CD8^+^ T cells, monocytes/macrophages (Ma), dendritic cells (DC), and neutrophils in the peripheral blood, spleen, and mLN, but there were no differences between mice in each group (Figure 3).

### Loss of TRIM59 in Macrophages Altered Both Local and Systemic Cytokines Following Sepsis

**Figure 4:**
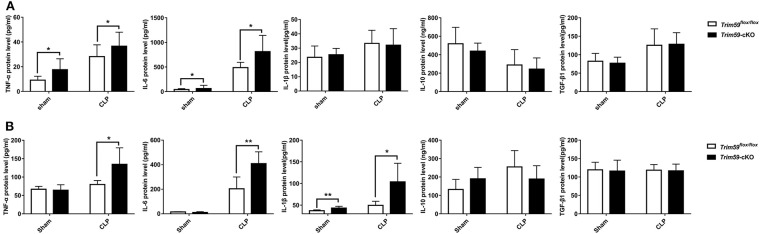
Loss of TRIM59 altered both local and systemic cytokines following sepsis. **(A)** The levels of TNF-α, IL-6, IL-1β, IL-10, and TGF-β1 were measured 24 h after surgery in the blood serum of *Trim59*^*flox*/*flox*^ and *Trim59*-cKO mice. **(B)** The levels of TNF-α, IL-6, IL-1β, IL-10, and TGF-β1 were measured 24 h after surgery in the BALFs of *Trim59*^*flox*/*flox*^ and *Trim59*-cKO mice. Sham group, *n* = 5, CLP group, *n* = 6. Data are presented as Means ± SEM, *p < 0.05, **p < 0.01.

In the above results, we observed that neutrophils were infiltrated in the peripheral blood of *Trim59*-cKO mice with sepsis, indicating that the inflammatory state of mice requires further investigation. Peripheral blood and bronchoalveolar lavage fluid (BALF) were obtained from CLP induced septic mice at 24 h after surgery. Expression levels of the inflammatory factor TNF-α, IL-6, IL-1β, IL-10, and TGF-β1 were measured to evaluate systematic and local immune status. The results demonstrated that the secretions of TNF-α and IL-6 were significantly increased after CLP surgery in the serum of *Trim59*-cKO mice, but there was no significant difference in IL-1β, IL-10, or TGF-β1 between *Trim59*^*flox*/*flox*^ mice and *Trim59*-cKO mice. Additionally, the expressions of TNF-α and IL-6 in *Trim59*-cKO mice were increased in the sham group, indicating that TRIM59 may have some anti-inflammatory effect at baseline (Figure 4A). In BALF, the infiltrations of TNF-α, IL-6, and IL-1β were upregulated after CLP surgery in *Trim59*-cKO mice, while IL-10 and TGF-β1 did not significantly change. Similar to what happened in plasma, the expression of IL-1β in *Trim59*-cKO mice was increased in the sham group (Figure 4B). In summary, the loss of TRIM59 exacerbated both the local and systemic inflammatory response in sepsis, and TRIM59 also played an anti-inflammatory role in resting state.

### Loss of TRIM59 in Macrophages Increased Bacterial Burden

**Figure 5:**
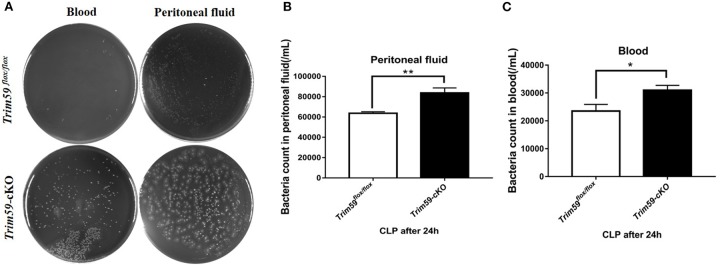
Loss of TRIM59 impacts bacterial burden. **(A)** Blood and peritoneal fluid were taken 24 h after the CLP surgery for bacterial culture. **(B)** Statistical comparison of bacteria counts in blood. **(C)** Statistical comparison of bacteria counts in peritoneal fluid. In each group, *n* = 6. Data are presented as Means ± SEM, *p < 0.05, **p < 0.01.

To explore the bacterial burden in *Trim59*^*flox*/*flox*^ mice and *Trim59*-cKO mice following CLP, we collected serum and peritoneal fluid 24 h after surgery which was then cultured for 24 h. The results showed that bacteria levels increased in *Trim59*-cKO mice both in the blood and in the peritoneal fluid (Figure 5).

### Loss of TRIM59 in Macrophages Suppressed Phagocytosis in Macrophages

**Figure 6:**
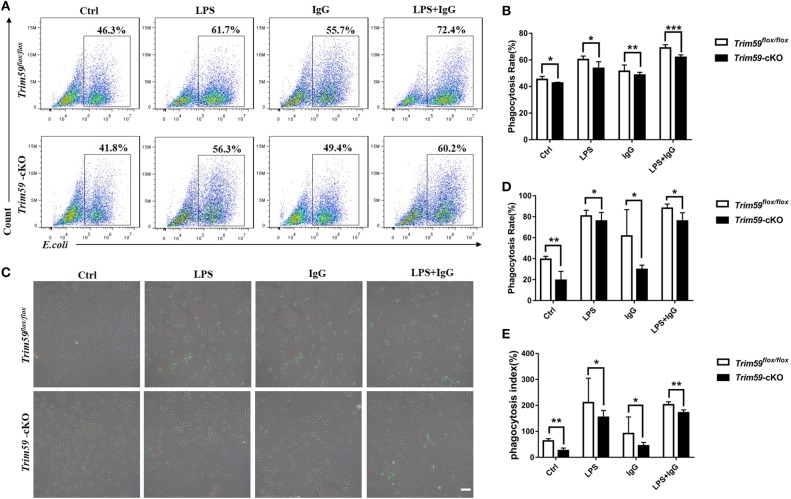
Loss of TRIM59 suppresses phagocytosis of macrophages. **(A)** BMDMs were incubated with *E. coli*, along with or without LPS or IgG, and the phagocytosis rates were measured using FACS. **(B)** Statistical comparison of phagocytosis rates. **(C)** The phagocytosis rates and phagocytosis index were measured using fluorescent microscope. **(D)** Statistical comparison of phagocyte rates. **(E)** Statistical comparison of phagocyte index. Original magnification × 200, scale bar = 50 μm. In each group, *n* = 6. Data are presented as Means ± SEM, *p < 0.05, **p < 0.01, ***p < 0.001.

Considering the powerful phagocytosis ability of macrophages, we explored the effect of TRIM59 on phagocytosis in macrophages. The structure of EGFP-vector and transfection efficiency are presented in Figure S4. We co-cultured BMDMs with LPS, IgG, and *E. coli* and found that the phagocytosis rate and phagocytosis index were decreased in TRIM59 knocked out BMDMs. This decrease also occurred when the BMDMs were co-cultured with LPS and/or IgG (Figure 6). Our results demonstrate that the phagocytosis of BMDMs decreased following TRIM59 deletion.

### Loss of TRIM59 in Macrophages Decreased Fcγ-Receptors in Macrophages

**Figure 7:**
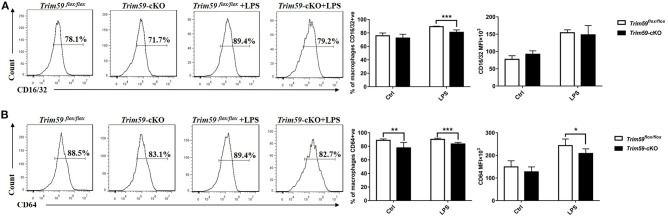
Loss of TRIM59 decreases Fcγ-receptors in macrophages. BMDMs were stimulated with or without LPS (0.2 μg/ml) for 24 h. After selecting all the F4/80^+^ cells, the expressions of CD16/32 **(A)** and CD64 **(B)** were measured by both percentage and MFI. In each group, *n* = 5 or *n* = 6. Data are presented as Means ± SEM, *p < 0.05, **p < 0.01, ***p < 0.001.

After confirming that the loss of TRIM59 suppressed phagocytosis in macrophages, especially IgG-mediated phagocytosis, we further explored whether TRIM59 affected Fcγ receptors. After selecting all F4/80^+^ cells, the expressions of CD16/32 and CD64 were significantly decreased in TRIM59-deleted BMDMs after LPS stimulation (Figure 7). We also examined the expression of co-stimulation receptors (MHCII, CD80, CD86), and no significantly change was observed (Figure S5).

### Loss of TRIM59 in Macrophages Regulated the Production of Cytokines in LPS-Stimulated BMDMs

**Figure 8:**
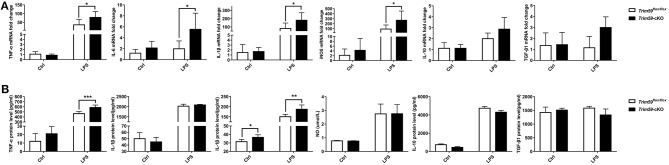
Loss of TRIM59 regulates the production of cytokines in LPS-stimulated BMDMs. **(A)** BMDMs were stimulated with or without LPS (0.2 μg/ml) for 24 h, and mRNA levels of TNF-α, IL-6, IL-1β, iNOS, IL-10, and TGF-β1were measured using qRT-PCR. **(B)** Protein level of TNF-α, IL-6, IL-1β, IL-10, and TGF-β1 were measured using Elisa and NO was measured using a kit. In each group, *n* = 5 or *n* = 6. Data are presented as Means ± SEM, *p < 0.05, **p < 0.01, ***p < 0.001.

We stimulated the BMDMs from both *Trim59*^*flox*/*flox*^ mice and *Trim59*-cKO mice with 0.2 μg/ml LPS for 18 h, and measured the expression of TNF-α, IL-6, IL-1β, iNOS, NO, IL-10, and TGF-β1. We found that the expression of TNF-α, IL-6, IL-1β, and iNOS were increased in TRIM59 knocked out BMDMs after LPS stimulation, but the expression of NO did not change. Additionally, the expression of IL-1β was upregulated in TRIM59 knocked out BMDMs without LPS stimulation, the expression of anti-inflammatory cytokines IL-10 and TGF-β1 did not change significantly (Figure 8). These results were consistent with the trend of previous *in vivo* experiments.

### Loss of TRIM59 in Macrophages Activated NF-κB Signaling Pathway

**Figure 9:**
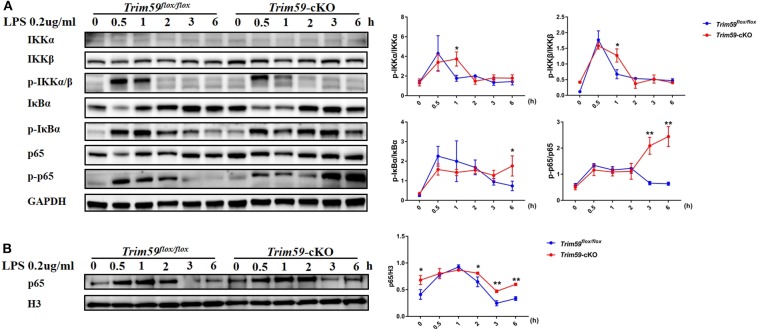
Loss of TRIM59 regulates the NF-κB signaling pathway. **(A)** BMDMs were stimulated with LPS (0.2 μg/ml) for the indicated times. The expression levels of IKKα, IKKβ, p-IKKα/β, IκBα, p- IκBα, p65, and p-p65 were measured using western blot analysis. Relative intensities were quantitated by densitometry using ImageJ and normalized to total protein levels; **(B)** The expression level of p65 in the nucleus was measured using western blot analysis. Relative intensities were quantitated by densitometry using ImageJ and normalized by H3 levels. Data are presented as Means ± SEM, *p < 0.05, **p < 0.01.

Previous results showed that TRIM59 could alter LPS-mediated inflammation, and activation and signaling through the NF-κB pathway is an important factor in LPS induced inflammation in macrophages. We stimulated BMDMs from *Trim59*^*flox*/*flox*^ mice and *Trim59*-cKO mice with 0.2 μg/ml LPS for 0, 0.5, 1, 2, 3, and 6 h, and found that p-IKKα/β was upregulated at 1 h after LPS stimulation in TRIM59-deleted BMDMs, and p-IκBα and p-p65 was upregulated at 3 and 6 h, respectively (Figure 9A). We also detected p65 in the nucleus when BMDMs were stimulated with 0.2 μg/ml LPS, and found that the expressions of p65 were increased as well. Notably, the expression of p65 in the nucleus was higher in the TRIM59-deleted BMDMs in the absence of LPS (Figure 9B). These results suggested that the removal of TRIM59 strengthened the LPS-induced inflammatory response via the NF-κB pathway, and TRIM59 also prevented p65 from entering the nucleus in the resting state. TRIM59 could interact with ECSIT and negatively regulate NF-κB (11), and ECSIT could stimulate IKKs through mitogen-activated protein kinase kinase kinase 1 (MAP3K1). ECSIT could also stimulate p65 directly (16), so we confirmed the expression of ECSIT and MAP3K1 in LPS-stimulated BMDMs, and found they did not significantly change (Figure S6A). Previous studies verified that TRIM59 was involved in the PI3K/AKT pathway (17–19), and that AKT could stimulate the NF-κB pathway via IKKs (20), but in our study, it seemed that the effect of TRIM59 in LPS-induced inflammation was independent of the PI3K/AKT signaling pathway (Figure S6B).

## Discussion

In our study, TRIM59 conditional knockout mice were bred to explore the effect of TRIM59 in myeloid derived macrophages in sepsis. We found that the expression of TRIM59 was significantly downregulated in LPS-stimulated BMDMs, and mortality, liver and lung damage, and inflammatory cytokines were increased in *Trim59*-cKO mice after CLP surgery. We also confirmed that bacterial burden greatly increased in *Trim59*-cKO mice following CLP surgery, and phagocytosis function and Fcγ receptors expression decreased when TRIM59 was knocked out in the BMDMs. Finally, we also demonstrated that secretions of pro-inflammatory cytokines were upregulated through the NF-κB signaling pathway in TRIM59-deleted BMDMs. In conclusion, we proved that TRIM59 in myeloid derived macrophages protected mice from sepsis by regulating inflammation and phagocytosis.

Our previous study found that TRIM59 promoted phagocytosis activity in the RAW264.7 cell line (21). Phagocytosis plays an important role in bacteria invasion, so we hypothesized that TRIM59 could affect sepsis by regulating macrophage phagocytosis. In order to prove our assumption, we examined bacteria burden following CLP surgery, and found that the bacteria burden was higher in *Trim59*-cKO mice. *In vitro* experiments, phagocytosis rate and the phagocytosis index were all used to evaluate the phagocytosis ability of macrophages. Phagocytosis rate indicates the average number of macrophages that phagocytic *E. coli* and phagocytosis index reflects the average number of ingested *E. coli*. Both of these indicators reflect the phagocytosis ability of macrophages. In our study, we found that phagocytosis ability was impaired in TRIM59 knocked out BMDMs. This phenomenon also occurred in the BMDMs co-cultured with LPS and/or IgG. All of these results indicated that TRIM59 could promote the phagocytosis of macrophages. In LPS-stimulated BMDMs, the expression of TRIM59 was downregulated, but phagocytosis ability was enhanced compared to unstimulated BMDMs, which suggested that TRIM59 is not the single decisive factor of phagocytosis. In LPS-stimulated BMDMs, the loss of TRIM59 attenuated the phagocytosis, which further demonstrated the role of TRIM59 in phagocytosis. There are many factors that affect the phagocytosis of macrophages, such as scavengers and integrins, including Fcγ receptors (22). Our further analysis indicated that Fcγ receptors were up-regulated by TRIM59, which confirmed the role of TRIM59 in macrophage phagocytosis in sepsis. However, the mechanisms underlying the regulation of Fcγ receptors by TRIM59 require additional investigation.

In LPS-stimulated macrophages, Toll-like receptor 4 (TLR4) recruits adapter proteins such as myeloid differentiation factor 88 (MyD88) to activate IL-1R-associated kinase (IRAK). When activated, IRAK induces the dephosphorylation of another adapter protein, receptor-associated factor TNF (TRAF6). In turn, TRAF6 interacts with the TGF beta-activated kinase 1 (TAK1) complex to phosphorylate I-κB kinases (IKKs). Inside the cytoplasm of an inactive macrophage IκB binds to NF-κB, leaving NF-κB in the cytoplasm. IKKs phosphorylates IκB, leading to degradation of IκB, and releasing NF-κB into the nucleus (23). ECSIT is an important protein in the NF-κB signal pathway. It can interact with MAP3K1 to activate p65 or activate p65 directly (16). Previous studies have demonstrated that TRIM59 can regulate pro-inflammation cytokines in macrophage cell lines (12), and regulate the innate immune response by interacting with ECSIT to affect the NF-κB and IRF3/7 signaling pathways (11). We hypothesized that TRIM59 would regulate secretion of cytokines by activating the NF-κB signaling pathway, which could aggravate sepsis. In both sham group mice and BMDMs without LPS stimulation, the expression of some pro-inflammatory cytokines increased, and the expression of p65 in nucleus was higher, indicating TRIM59 plays a role in inhibiting inflammation in macrophages, but the mechanism is unclear. We also showed that following LPS stimulation, p-p65 in TRIM59 knocked out BMDMs was abnormally activated, and the secretions of pro-inflammatory cytokines downstream were also abnormally high, which was exactly what we hypothesized. We also found that TRIM59 did not affect the expression of ECSIT and MAP3K1, which indicates that TRIM59 may activate the NF-κB signaling pathway through other mechanisms.

Current research focused on the function of TRIM59 in various types of cancer and has demonstrated that TRIM59 can regulate the PI3K/AKT signal pathway in various cancers. TRIM59 promotes neuroblastoma through the Wnt/beta-catenin (24) and PI3K/AKT signaling pathways (18), accelerates bladder cancer via the TGF-beta/Smad2/3 signaling pathway (25), and facilitates breast cancer (26), cholangiocarcinoma (17, 19), and ovarian cancer (27) through the PI3K/AKT signaling pathway. PI3K/AKT is also related to inflammation by activating IKKα and the whole NF-κB signal pathway (20). Based on these studies, it was worth further investigation whether TRM59 regulates sepsis through the NF-κB signaling pathway and/or the PI3K/AKT signaling pathway. In our study, we confirmed that p-p65 was upregulated in TRIM59 knocked out BMDMs, but the PI3K/AKT signaling pathway was not affected. Therefore, PI3K/AKT was not involved in the TRIM59 activated NF-κB signal pathway. Thus, TRIM59 could directly regulate inflammation via the NF-κB signal pathway, but the specific binding site or molecules involved requires further investigation.

TRIM proteins are defined as ubiquitin E3 ligase because of the RING figure (28). Many TRIM proteins take part in various physiopathologic processes by ubiquitination. For example, TRIM13 suppress TNF induced NF-κB activation by regulating NEMO ubiquitination (29). TRIM12c is involved in the Type I IFN and NF-κB pathways as an ubiquitin ligase (30). Zhou et al. reported that TRIM59 could aggravate the ubiquitination and degradation of p53 directly, thereby promoting gastric carcinogenesis (31). TRIM proteins participate in the NF-κB signaling pathway by ubiquitination, and as a member of TRIM protein family TRIM59 could also as an ubiquitin E3 ligase. In our study, we confirmed that TRIM59 regulated the activation of NF-κB, but whether TRIM59 functions as an ubiquitination remains unclear.

In conclusion, we demonstrated that expression of TRIM59 was significantly downregulated in LPS-stimulated BMDMs. Mortality, lung and liver damage, local and systemic inflammation were increased in *Trim59*-cKO mice with sepsis. Additionally, the depletion of TRIM59 in BMDMs upregulated the secretion of pro-inflammatory cytokines, and the NF-κB signaling pathway was involved in this regulation. We also found that bacteria loads were increased in *Trim59*-cKO mice with sepsis, and phagocytosis and Fcγ receptors were reduced in BMDMs from *Trim59*-cKO mice. The results of our study indicated that TRIM59 protected mice from sepsis by regulating inflammation and phagocytosis in macrophages.

## Data Availability Statement

The datasets generated for this study are available on request to the corresponding author.

## Ethics Statement

The animal study was reviewed and approved by Animal Research Committee of Jilin University.

## Author Contributions

ZJ, ZZ, and SL carried out all the experiments. ZJ, YH, MT, PZ, and YT analyzed the data and wrote the manuscript with support from DL, DY, and XZ. DY and XZ supervised the project. All authors approved the final version of the manuscript.

### Conflict of Interest

The authors declare that the research was conducted in the absence of any commercial or financial relationships that could be construed as a potential conflict of interest.
