# Supplementary material for: TRIM59 Protects Mice From Sepsis by Regulating Inflammation and Phagocytosis in Macrophages
Source: Front Immunol. 2020 Feb 18;11:263. doi: 10.3389/fimmu.2020.00263 (PMC7041419; doi:10.3389/fimmu.2020.00263)
Supplement: Supplementary file 1 [file Data_Sheet_1.docx]

**supplementary materials**


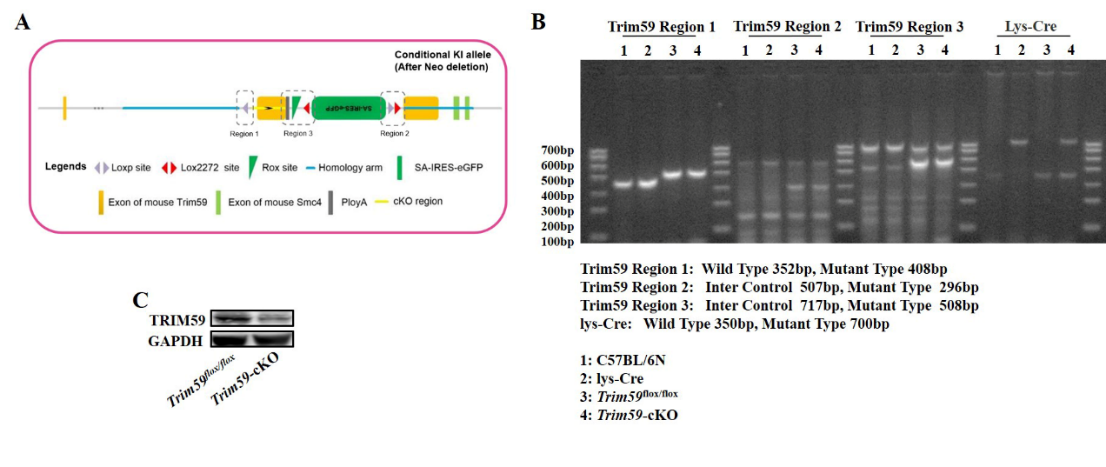


**Figure S1** Structure and identification of *Trim59^flox/flox^* and *Trim59*-cKO mice. (A) The structure of the loxp site. (B) Genotype identification of mice. (C) Protein level of TRIM59 in BMDMs from *Trim59^flox/flox^* and *Trim59*-cKO mice.


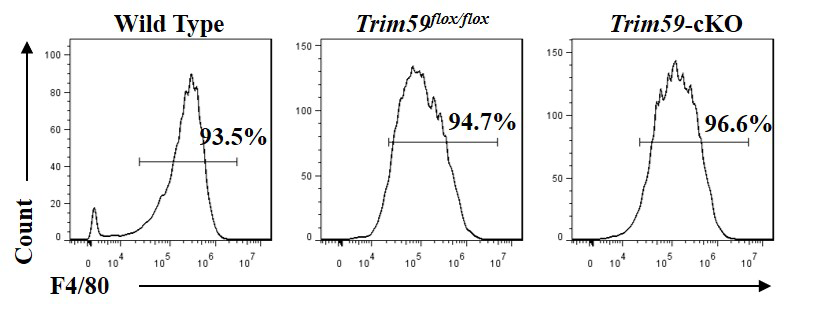


**Figure S2** Purities of BMDMs from C57BL/6N (wild type), *Trim59^flox/flox^* and *Trim59*-cKO mice.


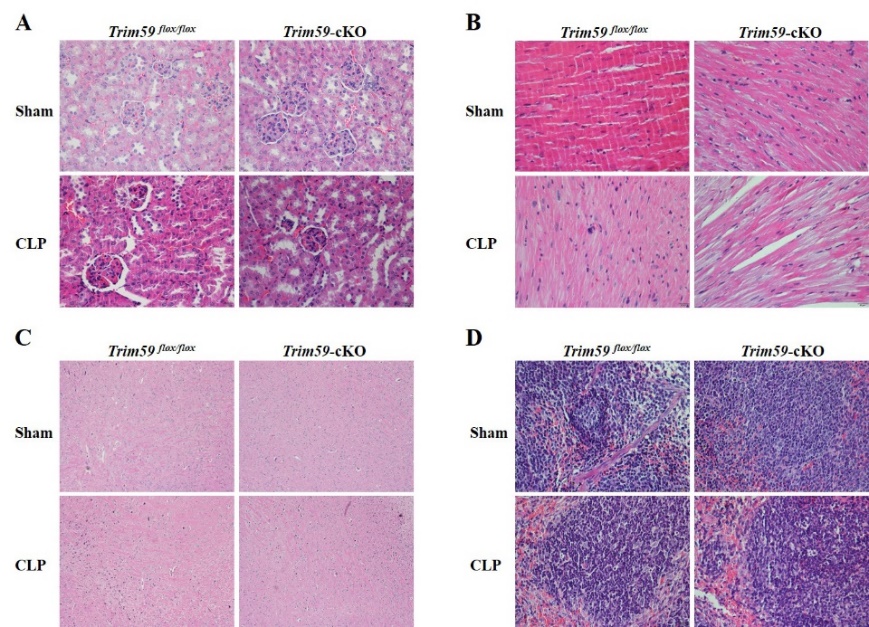


**Figure S3** Results of H&E staining of kidney (A), heart (B), brain (C), and spleen (D) tissues from *Trim59^flox/flox^* and *Trim59*-cKO mice after CLP for 24 hours. Original magnification ×400, scale bar = 20µm. Sham group, n=5, CLP group, n=6.


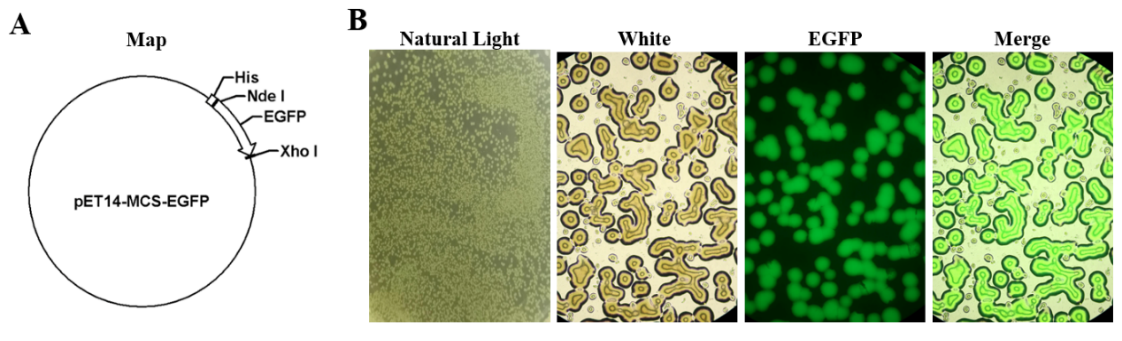


**Figure S4** Identification of *E.coli* with pET-14b-EGFP. (A) The structure of pET-14b-EGFP. (B) Transfection results.


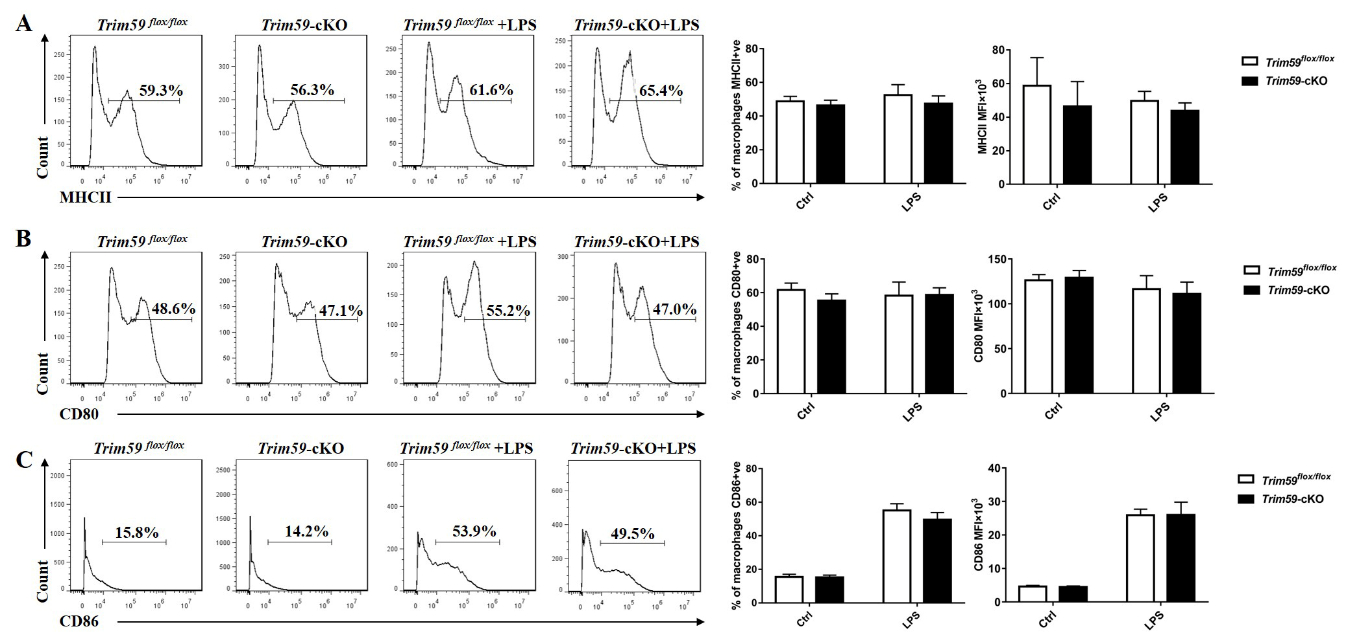


**Figure S5** BMDMs were stimulated for 24 hours with or without LPS (0.2μg/ml). After selecting all the F4/80^+^ cells, the expressions of co-stimulation proteins in LPS-stimulated BMDMs from *Trim59^flox/flox^* and *Trim59*-cKO mice: (A) MHCII, (B) CD80, and (C) CD86. In each group, n=5 or n=6. Data are presented as Means ± SEM.


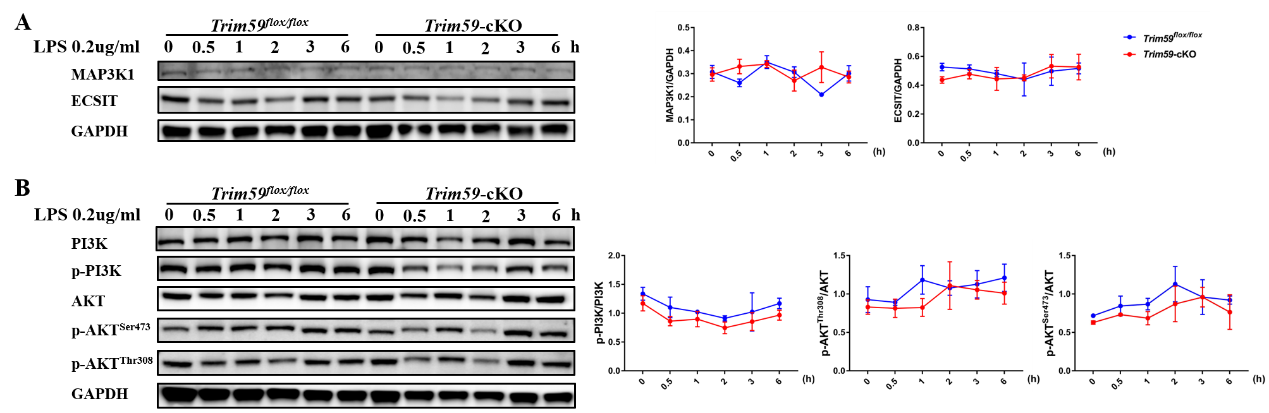


**Figure S6** BMDMs were stimulated with LPS (0.2μg/ml) for the indicated times. The expression of the MAP3K1, ECSIT (A) and PI3K/AKT signal pathways (B) in the LPS-stimulated BMDMs from *Trim59^flox/flox^* and *Trim59*-cKO mice. Relative intensities were quantitated by densitometry using ImageJ and normalized by total protein levels or by GAPDH. Data are presented as Means ± SEM.
